# Electronic health record-derived care-exposure burden and central line-associated bloodstream infection among critically ill adults: a multi-source retrospective cohort study

**DOI:** 10.3389/fpubh.2026.1883942

**Published:** 2026-06-24

**Authors:** Na Li, Jiali Huang, Jinglan Liu, Yang He

**Affiliations:** 1 Department of Rehabilitation Medicine, Yichang Central People’s Hospital, Yichang, Hubei, China; 2 Yichang Central People’s Hospital, Yichang, Hubei, China; 3 Department of Critical Care Medicine, Yichang Central People’s Hospital, The First College of Clinical Medical Science, China Three Gorges University, Yichang, Hubei, China

**Keywords:** care process, central line-associated bloodstream infection, critical care nursing, electronic health records, healthcare-associated infection, retrospective cohort study

## Abstract

**Background:**

Central line-associated bloodstream infection (CLABSI) remains a clinically important healthcare-associated infection in intensive care units. Routinely collected electronic health record (EHR) data may capture patient-level care-process complexity not reflected by conventional catheter-related variables alone.

**Objective:**

To examine whether an EHR-derived care-exposure burden score was associated with adjudicated CLABSI or CLABSI-like bloodstream infection among critically ill adults with central venous catheter exposure.

**Methods:**

This multi-source retrospective cohort study used harmonised data from MIMIC-IV, the eICU Collaborative Research Database, and a Chinese real-world critical care cohort. Adult patients with documented central venous catheter exposure and an ascertainable post-catheterisation observation window were included. The primary care-exposure burden score summarised cumulative care-process complexity using charting frequency, unique caregivers, concurrent vascular lines, catheter duration, pre-insertion mechanical ventilation duration, antibiotic-type count, and 24-h fluid input and output after catheterisation. Components were log-transformed, robustly standardised within each data source, averaged into a composite score, and categorised into database-specific quartiles. Multivariable logistic regression estimated adjusted odds ratios (aORs) and 95% confidence intervals (CIs). A 48-h landmark-compatible sensitivity analysis recalculated the score after excluding total catheter duration.

**Results:**

The final cohort included 12,693 patients: 10,868 from MIMIC-IV, 1,489 from eICU, and 336 from the Chinese cohort. Overall, 910 patients developed adjudicated CLABSI or CLABSI-like bloodstream infection (7.2%). Event rates were 4.7, 19.1, and 33.6%, respectively. Patients with events had higher care-exposure burden scores than those without events [median 0.27 (IQR, 0.00–0.46) vs. −0.02 (IQR, −0.21 to 0.20); *p* < 0.001]. In the primary clinically adjusted model, higher burden quartiles showed progressively greater odds compared with Q1: Q2 aOR 1.46, Q3 aOR 2.51, and Q4 aOR 4.99. The association was attenuated but persisted in the landmark-compatible analysis excluding total catheter duration.

**Conclusion:**

Higher EHR-derived care-exposure burden was associated with higher odds of adjudicated CLABSI or CLABSI-like bloodstream infection. The score should be interpreted as a pragmatic patient-level marker of cumulative care complexity and infection-prevention prioritisation, rather than as a causal measure of care quality or nursing performance. Prospective validation is required before clinical implementation.

## 1 Introduction

Central venous catheters are essential for haemodynamic monitoring, administration of vasoactive drugs, parenteral nutrition, renal replacement therapy, and complex fluid management in critically ill patients. Their use, however, exposes patients to central line-associated bloodstream infection (CLABSI), one of the most important device-associated healthcare infections monitored in hospital infection surveillance (1, 2). CLABSI is associated with septic shock, organ dysfunction, prolonged hospitalisation, higher healthcare costs, and excess mortality (3, 4). Although CLABSI is substantially preventable, it remains a persistent challenge for ICU infection prevention and nursing quality management (5).

Over the past two decades, CLABSI prevention has been shaped by evidence-based bundles that include hand hygiene, maximal sterile barrier precautions, chlorhexidine-based skin antisepsis, hub disinfection, dressing integrity, daily assessment of catheter necessity, and timely catheter removal (6, 7). Multicentre quality-improvement initiatives have shown that standardised insertion and maintenance practices can markedly reduce catheter-related bloodstream infections (8). Nevertheless, in real-world ICU settings, CLABSI risk is unlikely to be determined solely by insertion technique or isolated catheter characteristics. Illness severity, treatment complexity, care transitions, device burden, and workload pressure may all influence the feasibility of consistently maintaining optimal catheter care (9).

Existing evidence suggests that nurse understaffing, excessive workload, lower staffing levels, missed nursing care, and implementation barriers are associated with healthcare-associated infections and adverse patient outcomes (10–16). Nursing workload and documentation patterns may also reflect patient instability, monitoring intensity, and pressure within the care process (11, 17). However, most prior work has relied on single-centre data, unit-level staffing measures, or subjective workload instruments, limiting patient-level quantification of care-process exposure across multiple EHR data sources.

Electronic health records offer a practical opportunity to operationalise such exposure. Modern ICU EHR systems continuously capture timestamped information on vital signs, fluids, medications, devices, charting frequency, caregiver involvement, and key clinical events. Large public critical care databases such as MIMIC-IV and eICU, together with local real-world hospital data, allow the extraction of harmonised proxy indicators of care-contact intensity, care-team complexity, device burden, and treatment complexity (18, 19). These indicators are not direct measures of nursing workload or quality of care and should not be used to evaluate individual clinicians. They may, however, identify patients whose catheter maintenance occurs in a complex care environment requiring enhanced infection-prevention attention.

This study therefore constructed an EHR-derived care-exposure burden score using harmonised proxy indicators from MIMIC-IV, eICU, and a Chinese real-world critical care cohort, and examined its association with adjudicated CLABSI or CLABSI-like bloodstream infection among critically ill adults. We hypothesised that patients in higher care-exposure burden quartiles would have greater odds of the infection outcome and that this association would show a positive dose–response pattern.

## 2 Methods

### 2.1 Study design and reporting

This was a multi-source retrospective cohort study using routinely collected critical care data. The study was designed as an association study rather than a prediction model study. Reporting followed the STROBE statement and the RECORD extension for studies using routinely collected health data (20, 21).

### 2.2 Data sources

Two publicly available, de-identified critical care databases were used: MIMIC-IV and the eICU Collaborative Research Database. MIMIC-IV contains detailed clinical data from ICU admissions at Beth Israel Deaconess Medical Center between 2008 and 2022, and eICU contains multicentre ICU data from the United States collected between 2014 and 2015 (18, 19). The investigators completed the required data-use training and obtained database access certification. Because both databases are publicly available and de-identified, analyses of these datasets were exempt from additional institutional review.

The real-world Chinese cohort was derived from Yichang Central People’s Hospital. Local data were extracted using a dual-system linkage approach: catheter insertion and removal records were first identified through the hospital data-management system and then linked to the electronic medical record system to retrieve clinical characteristics. The local observation period extended from 1 January 2014 to 31 December 2023. Patients with clinically adjudicated CLABSI were identified through routine infection-control surveillance, and non-CLABSI patients with central venous catheter exposure were sampled from the same calendar period and similar ward contexts to reduce temporal and unit-level confounding. The local protocol was approved by the Ethics Committee of Yichang Central People’s Hospital (approval number: 2024–242-01), with waiver of written informed consent because of the retrospective and observational design.

### 2.3 Participants

Eligibility criteria were harmonised across the three data sources. Adult patients aged 18 years or older were included if they had an ICU or critical care unit admission record during the study period; a documented central venous catheter insertion or catheter exposure record during the ICU stay or ICU-related hospitalisation; an ascertainable catheterisation time; and at least one blood culture result available during the post-catheterisation observation period. For patients with multiple hospitalisations or ICU admissions, only the first eligible hospitalisation or ICU admission was retained to preserve statistical independence.

**Figure 1:**
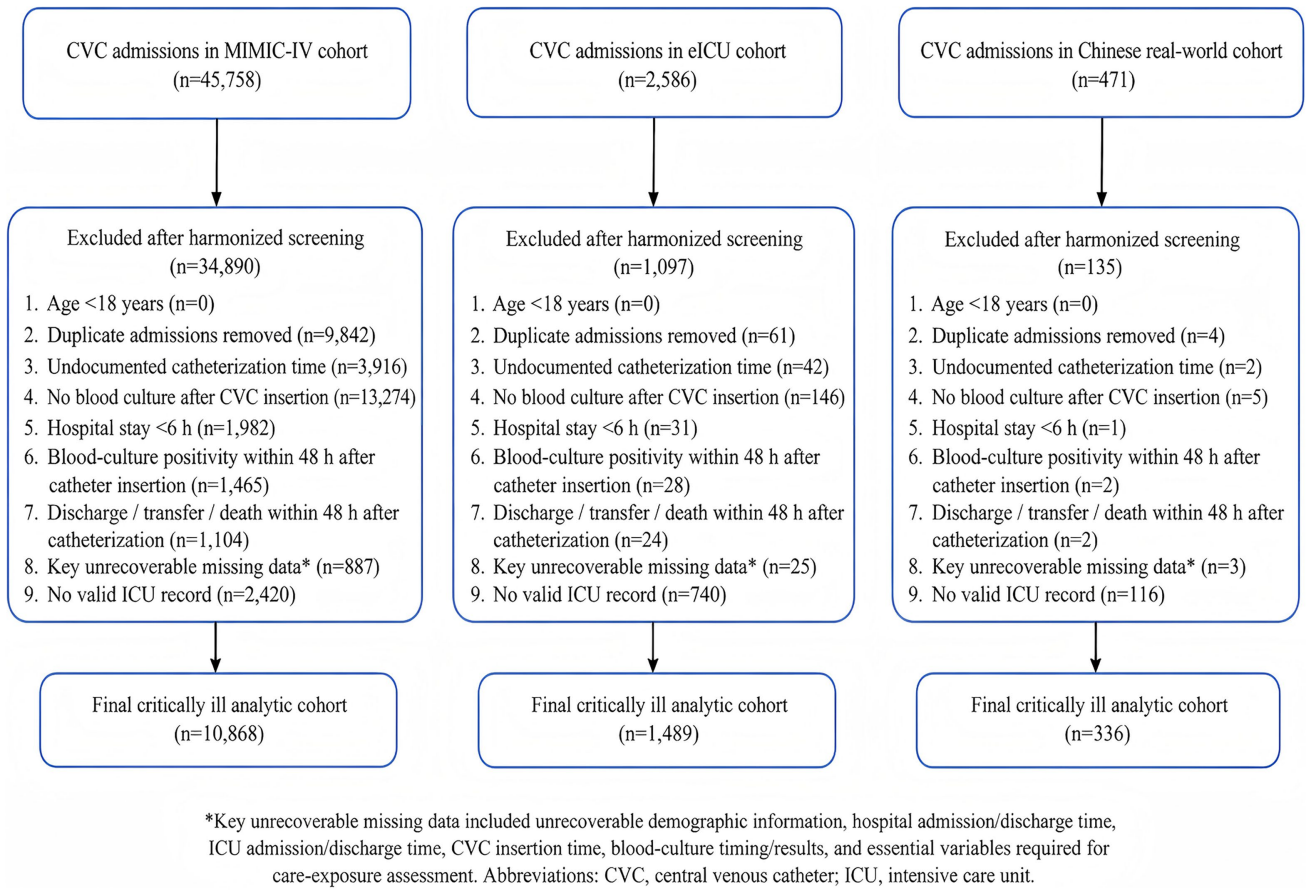
Study population selection flowchart.

Patients were excluded if key demographic or temporal variables, including age, sex, hospital admission or discharge time, ICU admission or discharge time, or catheterisation time, were missing and could not be reliably recovered; if the total hospital stay or ICU stay was too short to establish an evaluable observation window; if the catheter dwell time was shorter than 48 h and therefore precluded completion of the minimum catheter-related risk window; if blood culture positivity occurred within 48 h after catheter insertion; or if discharge, ICU transfer-out, interhospital transfer, or death occurred within 48 h after catheterisation and precluded completion of the minimum at-risk window. The detailed patient selection process for each data source is shown in Figure 1.

### 2.4 Exposure: EHR-derived care-exposure burden

The primary exposure was an EHR-derived care-exposure burden score. This score was not intended to directly measure nurse workload or to evaluate individual care quality. Instead, it was designed as a pragmatic proxy measure derived from routinely recorded EHR variables to summarise care-contact intensity, care-team complexity, catheter/device burden and treatment complexity within the catheter-related observation window. Candidate proxy indicators were prespecified before modelling according to clinical relevance, cross-source availability and temporal plausibility.

The final common indicators included daily charting frequency, number of unique caregivers, number of concurrent lines, catheter duration in days, ventilation hours before line placement, antibiotic-type count, fluid input during the 24 h after catheterisation and fluid output during the 24 h after catheterisation. These variables represented documentation/contact intensity, care-team complexity, catheter/device burden and treatment-complexity burden. Catheter duration was retained in the primary cumulative score because it represents the period during which catheter maintenance, line access, hub manipulation and dressing care may occur. However, because total catheter duration is also a direct risk factor for CLABSI and may contain post-landmark information, sensitivity analyses recalculated the score after excluding catheter duration.

The score was constructed in three steps. First, non-negative skewed component variables were transformed using log1p to reduce the influence of extreme values:

$$L_{\mathrm{ij}}=\log[1+\max(X_{\mathrm{ij}},0)]$$

where Xij denotes the raw value of proxy indicator j for patient i, and Lij denotes the transformed value. Second, transformed variables were robustly standardised within each database to reduce the influence of source-specific recording density and variable distributions across MIMIC-IV, eICU and the Chinese real-world cohort:

$$Z_{\mathrm{ij}}=[L_{\mathrm{ij}}-{\text{Median}}_{d}(L_{j})]/{\mathrm{IQR}}_{d}(L_{j})$$

where Mediand(Lj) and IQRd(Lj) denote the median and interquartile range of indicator j within database d. Finally, the patient-level overall care-exposure burden score was calculated as the mean of the available standardised proxy indicators:

$$\text{Care}-\text{exposure}\;{\text{burden score}}_{i}=(1/m_{i})\times \sum_{j}\;Z_{\mathrm{ij}}$$

where mi denotes the number of available component indicators for patient i. To improve interpretability and avoid direct comparison of absolute score values across databases, exposure quartiles were generated within each database, with Q1 representing the lowest burden and Q4 the highest burden. Domain scores for documentation/contact, catheter/device and treatment complexity were also constructed to explore the contribution of different care-process dimensions.

An equally weighted score was chosen *a priori* to preserve transparency, clinical interpretability and cross-database reproducibility. Data-driven weights, principal component analysis and regression-derived weights were not used because the aim was to construct a pragmatic care-complexity marker rather than an optimised prediction model. Regression-derived weights could also overfit to source-specific documentation practices and heterogeneous outcome ascertainment across databases.

To address temporal-ordering concerns, a 48-h landmark-compatible sensitivity analysis was performed using variables available within the fixed early post-catheterisation window or before catheter insertion. Total catheter duration was excluded from this modified score because duration up to 48 h was fixed by design among landmark-eligible patients, whereas total duration represented post-landmark information.

### 2.5 Outcome

The primary outcome was adjudicated CLABSI or CLABSI-like bloodstream infection. In MIMIC-IV and eICU, formally adjudicated surveillance-defined CLABSI could not be ascertained because infection-control adjudication, secondary-source attribution, catheter-maintenance documentation and organism-specific surveillance fields were not uniformly available. Therefore, events in the public databases were operationalised as CLABSI-like bloodstream infection using database-available EHR elements: documented central venous catheter exposure, ascertainable catheterisation time, at least one positive blood culture obtained more than 48 h after catheter insertion during the post-catheterisation observation window, and exclusion of blood-culture positivity within the first 48 h after catheterisation. When multiple positive blood cultures were present, the first qualifying positive culture was used as the event date. Because secondary-source attribution and organism-specific contaminant rules could not be applied uniformly, these outcomes were interpreted as broader CLABSI-like bloodstream infection rather than surveillance-confirmed CLABSI.

In the Chinese real-world cohort, CLABSI was adjudicated according to routine surveillance procedures aligned with CLABSI definitions. CVC exposure was verified using hospital catheter records, and suspected events were jointly reviewed by infectious disease/infection-control staff, the nursing department, the medical affairs department and treating clinicians. Identifiable alternative bloodstream-infection sources, likely contaminants and non-CLABSI explanations were excluded during adjudication. Operational details by data source are summarised in Supplementary Table S1.

### 2.6 Statistical analysis

Continuous variables are presented as medians and interquartile ranges, and categorical variables as counts and percentages. Baseline characteristics and care-exposure indicators were summarised by data source and by CLABSI status. Between-group differences were assessed using chi-square tests for categorical variables and non-parametric rank-sum or Kruskal-Wallis tests for continuous variables, as appropriate.

Multivariable logistic regression was used to estimate the association between EHR-derived care-exposure burden and adjudicated CLABSI or CLABSI-like bloodstream infection. Results are reported as adjusted odds ratios (aORs), 95% confidence intervals (CIs), and *p* values. Model 1 adjusted for care-exposure burden quartile, data source, age, and sex. Model 2 was prespecified as the primary model and additionally adjusted for available clinical status variables, including mean temperature, mean heart rate, mean arterial pressure, mean respiratory rate, minimum oxygen saturation, baseline white blood cell count, lactate, creatinine, blood urea nitrogen, platelet count, haemoglobin, and glucose, when variables met the predefined data-availability rule of less than 30% missingness and at least two distinct values. Model 3 replaced the composite burden quartile variable with the three domain scores while retaining the same data-source and clinical adjustment strategy. Variables potentially relevant to residual confounding but not uniformly available across all data sources are summarised in Supplementary Table S3.

For score construction, missing component values were not imputed; the composite score was calculated as the mean of available robustly standardised indicators. For regression covariates, variables with 30% or greater missingness or fewer than two distinct values were not entered into multivariable models, and complete cases were used for included covariates. Source-specific missingness patterns in the final analytical cohort are reported in Supplementary Table S2.

A trend analysis was performed by modelling the care-exposure burden quartile as an ordinal variable. A restricted cubic spline model with four knots was used to evaluate the adjusted dose–response association between the continuous care-exposure burden score and infection outcome, with the median score used as the reference value (22). Sensitivity analyses included source-specific models, Firth penalised logistic regression, E-value analysis for potential unmeasured confounding, additional adjustment for ICU and hospital lengths of stay, and the landmark-compatible no-duration score. Length-of-stay variables were treated as sensitivity covariates rather than primary covariates because they may lie on the causal or post-outcome pathway.

## 3 Results

### 3.1 Cohort composition and between-source differences

The final analytical cohort included 12,693 patients with central venous catheter exposure, comprising 10,868 patients from MIMIC-IV, 1,489 from eICU, and 336 from the Chinese real-world cohort. Overall, 910 patients developed adjudicated CLABSI or CLABSI-like bloodstream infection, corresponding to an event rate of 7.2%. Event rates differed substantially across data sources, increasing from 4.7% in MIMIC-IV to 19.1% in eICU and 33.6% in the Chinese cohort (Table 1).

**Table 1:** Baseline and care-exposure characteristics by data source.

**Characteristic**	**Overall** **(N = 12,693)**	**MIMIC-IV** **(N = 10,868)**	**eICU** **(N = 1,489)**	**Chinese real-world cohort** **(N = 336)**	**P value**
**CLABSI status**					**<0.001**
No CLABSI	11,783 (93%)	10,356 (95%)	1,204 (81%)	223 (66%)	
CLABSI	910 (7.2%)	512 (4.7%)	285 (19%)	113 (34%)	
Age, years	65 (54, 75)	65 (55, 75)	67 (54, 78)	67 (55, 77)	0.019
**Sex**					**<0.001**
Female	7,376 (58%)	6,631 (61%)	613 (41%)	132 (39%)	
Male	5,317 (42%)	4,237 (39%)	876 (59%)	204 (61%)	
Hospital length of stay, days	12 (7, 21)	12 (7, 22)	12 (7, 20)	15 (9, 26)	<0.001
ICU length of stay, days	5 (2, 11)	5 (2, 11)	6 (3, 12)	7 (4, 15)	<0.001
Catheter duration, days	2.9 (1.4, 6.1)	2.8 (1.3, 5.8)	4.2 (2.3, 8.1)	4.7 (2.3, 7.8)	<0.001
Daily EHR charting frequency, entries/day	72 (61, 90)	69 (60, 80)	670 (246, 1,381)	386 (161, 1,229)	<0.001
Number of unique caregivers	11 (7, 23)	14 (8, 26)	6 (4, 8)	5 (3, 7)	<0.001
Number of concurrent vascular lines	1.00 (1.00, 1.00)	1.00 (1.00, 1.00)	0.00 (0.00, 0.00)	0.00 (0.00, 1.00)	<0.001
Mechanical ventilation	9,881 (78%)	8,411 (77%)	1,293 (87%)	177 (53%)	<0.001
Ventilation duration before CVC insertion, h	6 (0, 69)	7 (0, 78)	4 (1, 23)	7 (1, 46)	0.004
Arterial catheter	8,321 (66%)	6,987 (64%)	1,140 (77%)	194 (58%)	<0.001
Dialysis	1,167 (9.2%)	1,158 (11%)	5 (0.3%)	4 (1.2%)	<0.001
Vasopressor use	6,322 (50%)	5,762 (53%)	466 (31%)	94 (28%)	<0.001
Antibiotic exposure within 48 h before CVC insertion	8,160 (64%)	6,576 (61%)	1,292 (87%)	292 (87%)	<0.001
Number of antibiotic agents	1.00 (0.00, 2.00)	1.00 (0.00, 2.00)	3.00 (2.00, 4.00)	3.00 (1.00, 4.00)	<0.001
Mean temperature, °C	36.89 (36.61, 37.25)	36.89 (36.64, 37.23)	36.89 (36.22, 37.44)	36.81 (36.26, 37.32)	<0.001
Mean heart rate, beats/min	85 (77, 96)	85 (77, 95)	87 (77, 99)	89 (77, 103)	<0.001
Mean arterial pressure, mm Hg	74 (68, 81)	73 (67, 80)	78 (71, 88)	78 (70, 87)	<0.001
Baseline white blood cell count, ×10^9/L	13 (10, 18)	14 (10, 19)	12 (9, 16)	12 (8, 17)	<0.001
White blood cell count at 48 h, ×10^9/L	14 (11, 19)	15 (11, 20)	11 (9, 15)	12 (8, 16)	<0.001
Serum lactate, mmol/L	2.40 (1.60, 3.60)	2.40 (1.70, 3.70)	2.00 (1.40, 3.10)	2.00 (1.40, 3.30)	<0.001
Serum creatinine, mg/dL	1.10 (0.80, 1.80)	1.10 (0.80, 1.90)	0.90 (0.70, 1.30)	1.20 (0.80, 2.30)	<0.001
Platelet count, ×10^9/L	203 (147, 284)	206 (150, 288)	187 (133, 254)	191 (126, 265)	<0.001
Fluid input within 24 h after CVC insertion, mL	6,189 (2,903, 11,558)	7,066 (3,114, 12,955)	4,411 (2,510, 7,086)	4,316 (2,544, 7,742)	<0.001
Fluid output within 24 h after CVC insertion, mL	2,407 (1,225, 3,670)	2,420 (1,198, 3,735)	2,450 (1,390, 3,500)	2,028 (1,013, 3,345)	0.003
Care-exposure burden score	0.00 (−0.21, 0.23)	0.00 (−0.20, 0.23)	−0.04 (−0.26, 0.21)	−0.01 (−0.23, 0.21)	<0.001
Care-exposure burden quartile					>0.90
Q1 (lowest)	3,174 (25%)	2,717 (25%)	373 (25%)	84 (25%)	
Q2	3,173 (25%)	2,717 (25%)	372 (25%)	84 (25%)	
Q3	3,173 (25%)	2,717 (25%)	372 (25%)	84 (25%)	
Q4 (highest)	3,173 (25%)	2,717 (25%)	372 (25%)	84 (25%)	

Values are presented as median (interquartile range) or n (%). *p* values were calculated using Pearson’s chi-square test for categorical variables and the Kruskal-Wallis rank-sum test for continuous variables. CLABSI indicates clinically adjudicated CLABSI in the Chinese cohort and CLABSI-like bloodstream infection in MIMIC-IV and eICU.

Baseline clinical characteristics, treatment exposures, and care-process indicators also varied significantly among the three cohorts. Patients in eICU and the Chinese cohort generally had longer catheter duration, higher EHR charting frequencies, and greater antibiotic exposure before catheter insertion than those in MIMIC-IV. These differences supported within-database standardisation of exposure components and adjustment for data source in pooled regression models.

### 3.2 Care-exposure burden according to infection status

Patients who developed adjudicated CLABSI or CLABSI-like bloodstream infection showed a consistently higher care-exposure burden than those without events. They had longer catheter duration, higher daily EHR charting frequency, more unique caregivers, longer pre-insertion mechanical ventilation duration, and greater exposure to arterial catheters, dialysis, and antibiotics (Table 2).

**Table 2:** Characteristics according to CLABSI status.

Characteristic	No CLABSI (*N* = 11,783)	CLABSI (*N* = 910)	*P* value
Data source			<0.001
MIMIC-IV	10,356 (88%)	512 (56%)	
eICU	1,204 (10%)	285 (31%)	
Chinese real-world cohort	223 (1.9%)	113 (12%)	
Age, years	66 (55, 76)	62 (48, 72)	<0.001
Sex			<0.001
Female	6,933 (59%)	443 (49%)	
Male	4,850 (41%)	467 (51%)	
Catheter duration, days	2.8 (1.3, 5.5)	8.2 (5.3, 12.7)	<0.001
Daily EHR charting frequency, entries/day	72 (61, 88)	80 (63, 739)	<0.001
Number of unique caregivers	11 (7, 22)	19 (7, 38)	<0.001
Number of concurrent vascular lines	1.00 (1.00, 1.00)	1.00 (0.00, 1.00)	<0.001
Mechanical ventilation	9,090 (77%)	791 (87%)	<0.001
Ventilation duration before CVC insertion, h	4 (0, 59)	93 (8, 355)	<0.001
Arterial catheter	7,605 (65%)	716 (79%)	<0.001
Dialysis	983 (8.3%)	184 (20%)	<0.001
Vasopressor use	5,883 (50%)	439 (48%)	0.3
Antibiotic exposure within 48 h before CVC insertion	7,413 (63%)	747 (82%)	<0.001
Number of antibiotic agents	1.00 (0.00, 2.00)	3.00 (1.00, 4.00)	<0.001
Mean temperature, °C	36.87 (36.61, 37.22)	37.10 (36.67, 37.55)	<0.001
Mean heart rate, beats/min	85 (76, 95)	91 (81, 101)	<0.001
Mean arterial pressure, mm Hg	74 (68, 81)	76 (69, 84)	<0.001
Baseline white blood cell count, ×10^9/L	13 (10, 18)	15 (11, 21)	<0.001
White blood cell count at 48 h, ×10^9/L	14 (11, 19)	15 (11, 22)	<0.001
Serum lactate, mmol/L	2.40 (1.60, 3.60)	2.50 (1.50, 4.40)	0.077
Serum creatinine, mg/dL	1.10 (0.80, 1.80)	1.30 (0.80, 2.70)	<0.001
Platelet count, ×10^9/L	201 (147, 280)	228 (146, 354)	<0.001
Fluid input within 24 h after CVC insertion, mL	6,282 (2,912, 11,580)	5,123 (2,770, 11,262)	0.14
Fluid output within 24 h after CVC insertion, mL	2,435 (1,254, 3,702)	2,098 (1,071, 3,400)	<0.001
Documentation/contact score	0.04 (−0.29, 0.31)	0.30 (−0.06, 0.55)	<0.001
Catheter/device score	0.01 (−0.21, 0.33)	0.44 (0.20, 0.67)	<0.001
Treatment-complexity score	−0.09 (−0.46, 0.20)	0.13 (−0.20, 0.42)	<0.001
Care-exposure burden score	−0.02 (−0.21, 0.20)	0.27 (0.00, 0.46)	<0.001
Care-exposure burden quartile			<0.001
Q1 (lowest)	3,072 (26%)	102 (11%)	
Q2	3,060 (26%)	113 (12%)	
Q3	2,970 (25%)	203 (22%)	
Q4 (highest)	2,681 (23%)	492 (54%)	

Values are presented as median (interquartile range) or n (%). *P* values were calculated using Pearson’s chi-square test for categorical variables and the Wilcoxon rank-sum test for continuous variables. CLABSI indicates clinically adjudicated CLABSI in the Chinese cohort and CLABSI-like bloodstream infection in MIMIC-IV and eICU.

**Figure 2:**
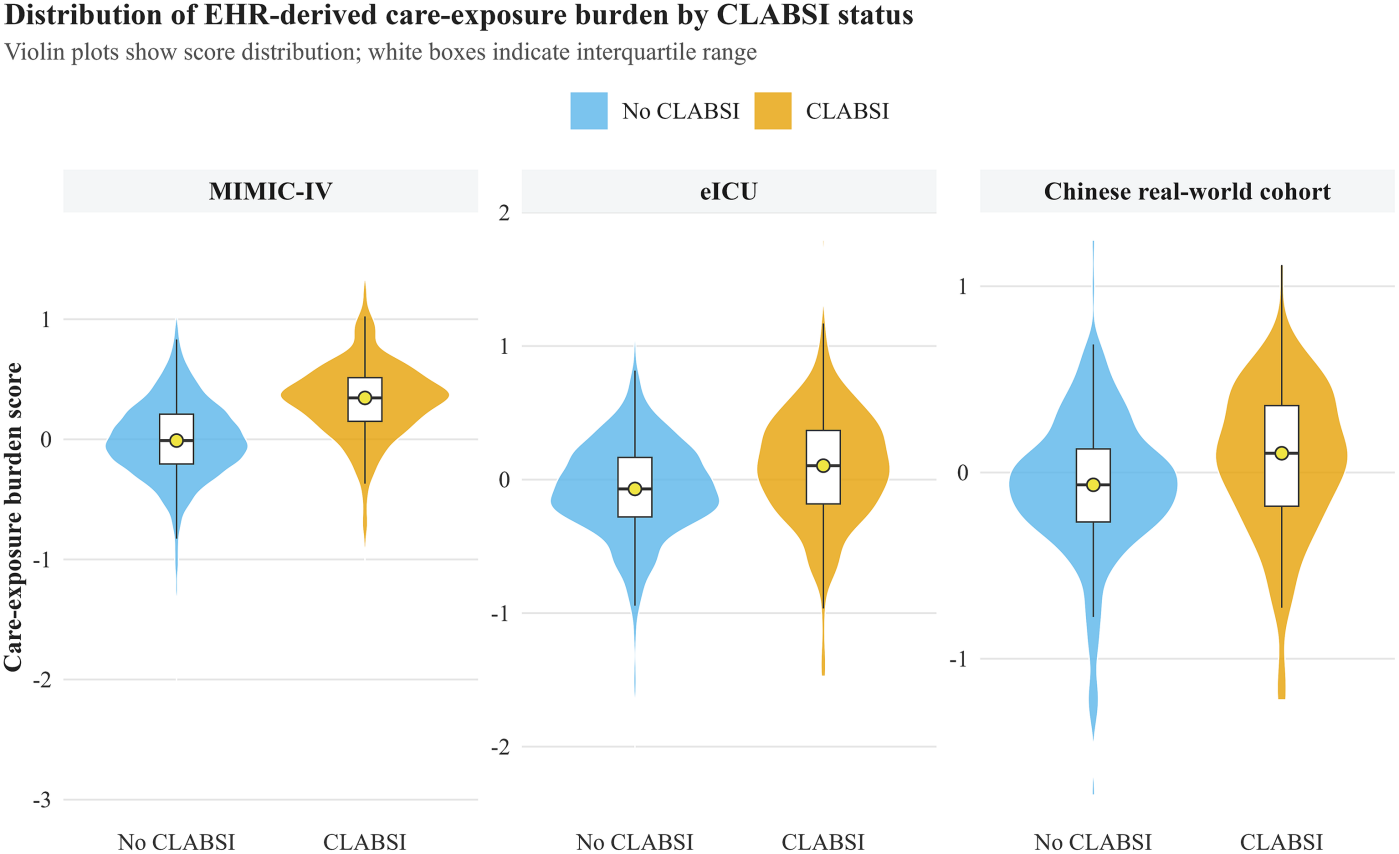
Distribution of EHR-derived care-exposure burden by CLABSI status. Violin plots show score distributions; white boxes indicate interquartile ranges.

The overall care-exposure burden score was higher in the event group than in the non-event group [0.27 (IQR, 0.00–0.46) vs. − 0.02 (IQR, −0.21 to 0.20); *p* < 0.001]. Similar differences were observed for the documentation/contact, catheter/device, and treatment-complexity domain scores (Table 2 and Figure 2). A heatmap of standardised component values is provided in Supplementary Figure S1. More than half of events occurred in the highest care-exposure burden quartile, whereas only 11% occurred in the lowest quartile, indicating a marked concentration of events among patients with the highest EHR-derived burden.

### 3.3 Crude event gradient across care-exposure burden quartiles

**Figure 3:**
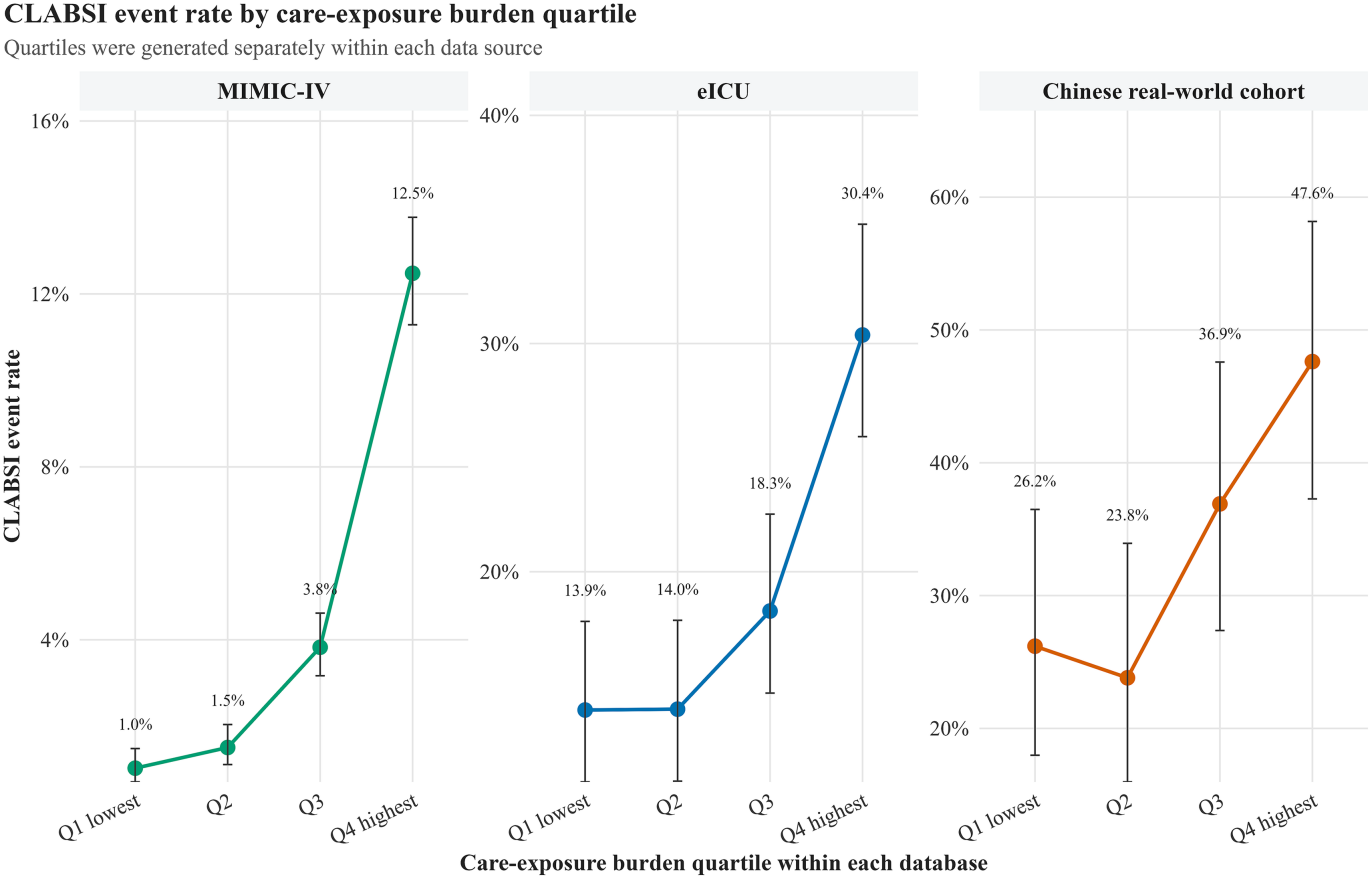
Crude event rates across care-exposure burden quartiles. Event rates are shown across database-specific quartiles of EHR-derived care-exposure burden. Points indicate crude event rates, and error bars indicate Wilson 95% confidence intervals.

Crude event rates showed an overall increasing pattern across higher care-exposure burden quartiles within each data source (Figure 3 and Supplementary Table S4). The gradient was most apparent in MIMIC-IV, where the event rate increased from 1.0% in Q1 to 12.9% in Q4, and in eICU, where it increased from 14.5 to 27.4%. In the Chinese real-world cohort, event rates were higher overall and rose from 26.2% in Q1 to 47.6% in Q4. Decile-based descriptive analyses showed a similar within-database risk gradient (Supplementary Figure S2).

### 3.4 Adjusted association between care-exposure burden and infection outcome

**Figure 4:**
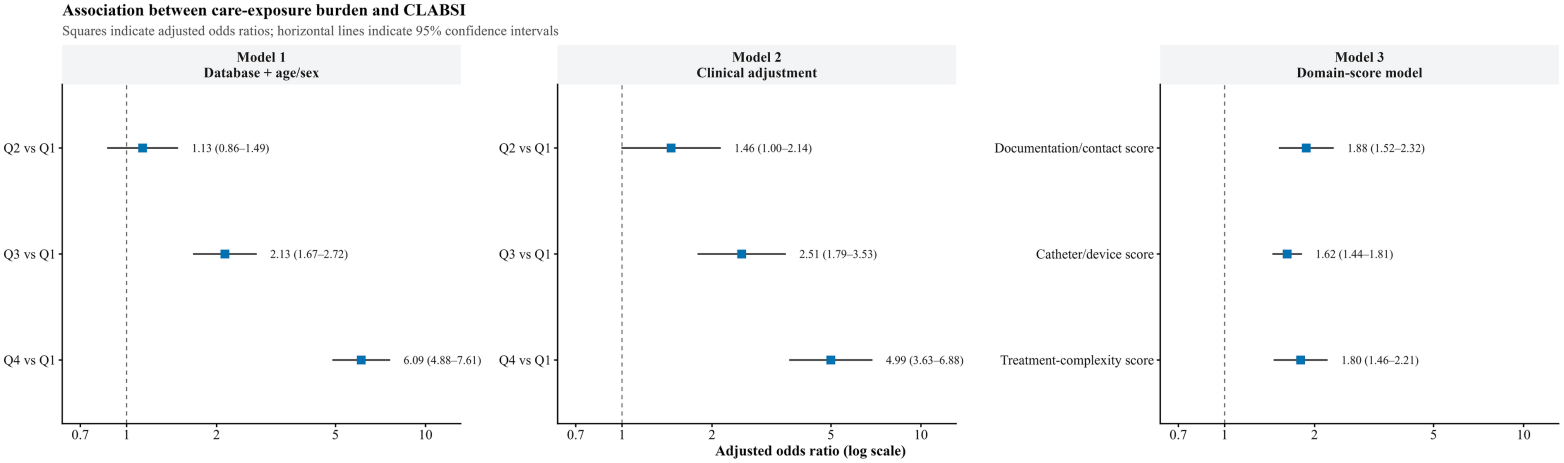
Adjusted association between care-exposure burden and CLABSI or CLABSI-like bloodstream infection. Squares indicate adjusted odds ratios and horizontal lines indicate 95% confidence intervals. The vertical dashed line denotes an odds ratio of 1.0. Model 1 adjusted for data source, age, and sex. Model 2 additionally adjusted for available clinical status variables. Model 3 replaced the composite burden quartile variable with the three domain scores.

Higher EHR-derived care-exposure burden was associated with progressively greater odds of adjudicated CLABSI or CLABSI-like bloodstream infection in multivariable models (Figure 4). In the primary clinically adjusted model, compared with Q1, the aORs were 1.46 (95% CI, 1.00–2.14; *p* = 0.053) for Q2, 2.51 (95% CI, 1.79–3.53) for Q3, and 4.99 (95% CI, 3.63–6.88) for Q4. The association was strongest in the highest burden quartile and remained evident after adjustment for data source, demographic characteristics, and available clinical status variables.

When the composite burden score was replaced by domain scores, all three domains were positively associated with the infection outcome. The aORs were 1.88 (95% CI, 1.52–2.32) for the documentation/contact score, 1.62 (95% CI, 1.44–1.81) for the catheter/device score, and 1.80 (95% CI, 1.46–2.21) for the treatment-complexity score. These findings suggest that the association reflected multiple dimensions of care contact, device burden, and treatment complexity.

### 3.5 Dose–response association between care-exposure burden and infection outcome

**Figure 5:**
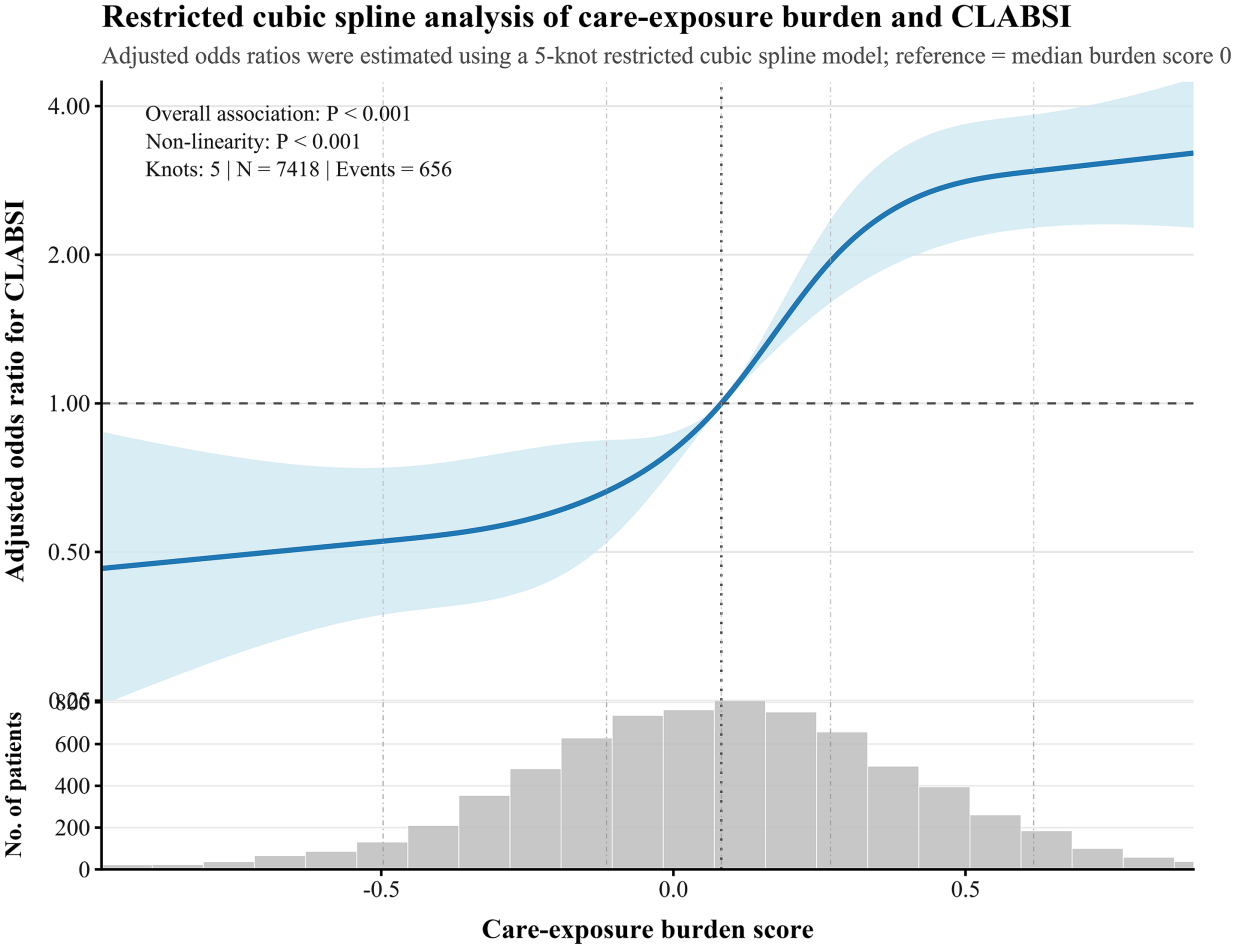
Restricted cubic spline analysis of the association between care-exposure burden score and CLABSI or CLABSI-like bloodstream infection. The solid line indicates the adjusted odds ratio, and the shaded area indicates the 95% confidence interval. The median care-exposure burden score was used as the reference. The horizontal dashed line denotes an odds ratio of 1.0, and the vertical dotted line indicates the reference value.

Restricted cubic spline analysis was conducted in the pooled cohort to explore the shape of the association between the continuous EHR-derived care-exposure burden score and infection outcome. Using a four-knot spline model with the median burden score as the reference, the adjusted curve showed a positive non-linear dose–response pattern (Figure 5). The odds of infection increased progressively as the burden score rose above the reference range, with a steeper increase across the middle-to-high range and a tendency to plateau at the highest values. Confidence intervals widened at the extremes, where fewer observations were available. This pattern was consistent with the quartile-based analyses.

### 3.6 Sensitivity and supporting analyses

Sensitivity and supporting analyses were broadly consistent with the primary findings. In source-specific models, the highest care-exposure burden quartile remained associated with higher odds of adjudicated CLABSI or CLABSI-like bloodstream infection in MIMIC-IV, eICU, and the Chinese real-world cohort, although the magnitude of association varied across data sources (Supplementary Table S5). Catheter type and insertion-site distributions in data sources with available catheter-characteristic information are provided in Supplementary Table S6. A significant linear trend was observed across increasing burden quartiles when the exposure was modelled as an ordinal variable (OR per quartile increase, 1.78; 95% CI, 1.62–1.96; P for trend <0.001; Supplementary Table S7). Model diagnostics are reported descriptively in Supplementary Table S8 and are not intended to represent prediction-model validation. The results were robust in Firth penalised logistic regression (Supplementary Table S9). Additional adjustment for ICU and hospital length of stay attenuated the estimates but preserved the positive graded association for Q3 and Q4 (Supplementary Table S10 and Supplementary Figure S5). The E-value for the Q4 versus Q1 association was 9.20 for the point estimate and 6.58 for the confidence-limit closest to the null (Supplementary Table S11).

In the landmark-compatible sensitivity analysis excluding total catheter duration, higher modified burden quartiles remained associated with the infection outcome in pooled clinically adjusted models, although effect estimates were attenuated compared with the primary cumulative score (Q2 aOR 1.46, 95% CI 1.05–2.04; Q3 aOR 1.77, 95% CI 1.30–2.41; Q4 aOR 3.20, 95% CI 2.40–4.25; Supplementary Tables S12–S14 and Supplementary Figure S3). Source-specific no-duration analyses showed persistence of the gradient in MIMIC-IV, with greater attenuation in eICU and the Chinese cohort (Supplementary Table S15 and Supplementary Figure S4). These results indicate that prolonged catheter exposure contributed to the cumulative-score association but did not fully account for the pooled association.

## 4 Discussion

In this multi-source retrospective cohort study of 12,693 critically ill adults with central venous catheter exposure, higher EHR-derived care-exposure burden was associated with adjudicated CLABSI or CLABSI-like bloodstream infection. Patients who developed events had higher overall care-exposure burden scores and greater exposure across documentation/contact, catheter/device, and treatment-complexity domains. In the primary clinically adjusted model, the odds increased progressively across burden quartiles, with the highest quartile showing approximately five-fold higher adjusted odds compared with the lowest quartile. Restricted cubic spline analysis suggested a non-linear dose–response pattern. These findings indicate that the score captures patient-level care complexity during the catheter-at-risk period, but they should not be interpreted as evidence of causal effects of the individual score components.

Our findings extend the existing CLABSI literature in several important ways. Current prevention frameworks emphasise insertion technique, maximal sterile barrier precautions, chlorhexidine-based skin antisepsis, hub disinfection, dressing integrity, daily review of catheter necessity, and timely catheter removal (6, 7). These measures remain the foundation of CLABSI prevention, and large-scale quality-improvement initiatives have shown that standardised bundles can substantially reduce catheter-related bloodstream infections (8). However, despite these advances, CLABSI continues to occur in ICUs and acute-care settings, particularly among patients with prolonged device exposure and high treatment complexity (1–4). Recent multicentre surveillance and national/regional meta-analytic data further show that CLABSI epidemiology varies markedly across healthcare systems, ICU types, surveillance intensity, and catheter-utilisation patterns (23, 24). The present study suggests that routinely captured EHR signals may help identify patients in whom sustaining optimal catheter maintenance is practically more difficult because of frequent documentation, multiple caregivers, longer catheter duration, concurrent devices, antimicrobial exposure, mechanical ventilation, and intensive fluid management.

A key contribution of this study is the operationalisation of care-exposure burden at the patient level. Prior studies have linked nurse understaffing, excessive workload, lower staffing levels, and missed nursing care with healthcare-associated infections and adverse outcomes (10–15). More recent nursing-focused evidence also indicates that knowledge gaps, perceived barriers, workload pressure, and protocol availability can influence adherence to CLABSI prevention practices among critical care nurses (16). Nevertheless, much of the existing evidence has relied on unit-level staffing indicators, self-reported workload measures, or hospital-level administrative data. These approaches are valuable but may not fully capture patient-specific care processes occurring during the central-line exposure window. By contrast, the present study used EHR-derived patient-level proxy measures to summarise care-contact intensity, team complexity, device burden, and treatment complexity across three heterogeneous data sources.

The observed association should not be interpreted as evidence that documentation intensity, caregiver involvement, antibiotic exposure or ventilation duration causes CLABSI. More frequent documentation may reflect patient instability, closer monitoring, or heightened clinical concern rather than unnecessary care. Similarly, a larger number of caregivers may indicate multidisciplinary management, complex procedures, prolonged ICU exposure, or repeated transitions in bedside responsibility. The care-exposure burden score is therefore best understood as a pragmatic EHR-derived marker of patient-level care complexity and infection-prevention prioritisation, rather than as a causal measure of care quality, nursing performance, or individual clinician practice.

Catheter duration deserves particular caution because it is both a component of cumulative catheter/device burden and a well-established risk factor for CLABSI. Its inclusion in the primary score is clinically meaningful for a retrospective cumulative burden marker, but total duration is not suitable for a fixed early-landmark exposure because it is only fully known after subsequent follow-up. The landmark-compatible analysis excluding total catheter duration attenuated the association but preserved a pooled graded pattern. This finding suggests that line-days contribute importantly to the original cumulative-score association, while other EHR-derived care-exposure indicators also carry information about infection risk.

Several plausible mechanisms may explain the graded association. Longer catheter duration increases the period during which microbial contamination, hub manipulation, dressing disruption, or biofilm formation may occur (7, 9). Critically ill patients with multiple devices and intensive therapies are exposed to frequent line access for medications, blood sampling, fluid resuscitation, vasoactive drugs, renal replacement therapy, parenteral nutrition, and antimicrobial administration. Greater documentation/contact intensity and a larger number of unique caregivers may signal repeated handoffs and fragmented task ownership, increasing the need for explicit communication about line necessity, dressing status, access frequency, and maintenance responsibilities. Finally, high treatment complexity may be a marker of illness severity incompletely captured by routinely available physiological variables.

The consistency of the crude gradient across MIMIC-IV, eICU, and the Chinese real-world cohort should be interpreted in the context of substantial outcome and data-source heterogeneity. Absolute event rates should not be directly compared across datasets because of differences in patient selection, microbiological testing practices, surveillance intensity, case mix, catheter utilisation, local infection-control workflows, and outcome ascertainment. Public databases relied on an operational CLABSI-like definition derived from available EHR elements, whereas the Chinese cohort used clinically adjudicated CLABSI surveillance. The more important finding is that higher within-database care-exposure burden was associated with greater infection risk after database-specific standardisation and adjustment for data source.

This study also contributes to the growing field of EHR-enabled infection surveillance and risk stratification. Large critical care databases such as MIMIC-IV and eICU have enabled reproducible research using granular physiological, treatment, microbiological, and process-of-care data (18, 19). Automated surveillance systems for healthcare-associated infections have been increasingly explored, although implementation, interoperability, and diagnostic reliability vary across settings (25). Recent work on EHR-based CLABSI prediction has highlighted the importance of time-updated modelling and competing events when using routinely recorded hospital data (26), and business-intelligence approaches suggest that electronic triggers combined with expert review may support proactive catheter reassessment and infection-prevention workflows (27). More broadly, real-world implementation of AI-enabled infection-prevention tools highlights that model development must be followed by workflow integration, governance, user acceptance and assessment of measurable effects on infection outcomes (28). Our score does not replace formal surveillance definitions or manual infection-control adjudication; it is best viewed as an interpretable EHR-derived signal for prioritising prevention review.

In practice, the score could be implemented as a non-punitive EHR-derived signal reviewed by infection-prevention teams, ICU charge nurses, or catheter-maintenance teams. Potential actions may include structured catheter-necessity reassessment, targeted dressing and hub-access review, bedside handoff prompts, and focused line-maintenance audit-feedback for patients with high cumulative care complexity. The score should not by itself trigger empirical antimicrobial therapy, routine blood-culture testing, or invasive procedures. Implementation should also monitor unintended consequences, including alert fatigue, additional documentation burden, over-surveillance, and inappropriate attribution of infection risk to individual clinicians or nursing teams. This implementation pathway is consistent with evidence that layered quality-improvement initiatives, rather than isolated alerts, are needed to reduce CLABSI in complex ICU settings (29).

The strengths of this study include its multi-source design, large sample size, inclusion of both public ICU databases and a real-world Chinese cohort, harmonised eligibility criteria, database-specific robust standardisation of exposure components, and evaluation of composite, domain-specific and no-duration sensitivity scores. The restricted cubic spline analysis further strengthened interpretation by showing that the association was not merely an artefact of quartile categorisation.

Future research should prospectively validate this score in contemporary ICU cohorts with richer catheter-maintenance data, direct nurse staffing information, device utilisation ratios, bundle adherence, catheter insertion site, catheter type, lumen number, dressing condition, line-access frequency, and microbiological adjudication. The score should also be evaluated as a dynamic, time-updated measure because care exposure and competing clinical events change continuously during hospitalisation (26). Prospective implementation studies should determine whether adding care-exposure burden signals to infection-prevention dashboards improves catheter review, reduces unnecessary line days, or decreases CLABSI incidence without increasing alarm fatigue or documentation burden.

This study has several limitations. First, because of its retrospective observational design, causal inference cannot be established, and residual confounding may remain despite adjustment for data source, demographic characteristics, and available clinical status variables. Harmonised severity scores, comorbidity burden, immunosuppression, malignancy, parenteral nutrition, catheter type, insertion site, number of lumens, emergency insertion, unit-level workload and CLABSI bundle adherence were not uniformly available across all three data sources. Therefore, part of the observed association may reflect unmeasured severity of illness, invasive-treatment intensity, device complexity, or local infection-prevention practice rather than modifiable care-process exposure. Second, the care-exposure burden score was derived from EHR-based proxy indicators rather than direct measurements of nursing workload, staffing adequacy, catheter-maintenance quality, or bundle adherence. Third, outcome ascertainment differed across data sources. The Chinese cohort used clinically adjudicated CLABSI surveillance, whereas MIMIC-IV and eICU relied on an operational CLABSI-like definition based on available EHR elements; misclassification remains possible. Fourth, heterogeneity in EHR systems, documentation practices, microbiological testing, patient case mix, and infection-control workflows may limit direct comparability across cohorts. Finally, the clinical utility of the score has not yet been prospectively evaluated.

## 5 Conclusion

In this multi-source cohort of critically ill adults with central venous catheter exposure, higher EHR-derived care-exposure burden was associated with higher odds of adjudicated CLABSI or CLABSI-like bloodstream infection. The association was graded across burden quartiles, supported by domain-specific analyses, and partly persisted after excluding total catheter duration in a landmark-compatible sensitivity analysis. The score should be interpreted as a pragmatic marker of patient-level care complexity and infection-prevention prioritisation, rather than as a causal measure of care quality, nursing performance, or individual clinician practice. Prospective validation with harmonised surveillance definitions and workflow-integrated implementation studies is needed before routine clinical use.

## Data Availability

The original contributions presented in the study are included in the article/Supplementary material, further inquiries can be directed to the corresponding author.
