# Supplementary material for: Electronic health record-derived care-exposure burden and central line-associated bloodstream infection among critically ill adults: a multi-source retrospective cohort study
Source: Front Public Health. 2026 Jun 24;14:1883942. doi: 10.3389/fpubh.2026.1883942 (PMC13341585; doi:10.3389/fpubh.2026.1883942)
Supplement: Supplementary file 1 [file Supplementary_file_1.DOCX]

**Supplementary Material**

**Supplementary Tables**

**Supplementary Table S1. Operational definition and ascertainment of CLABSI or CLABSI-like bloodstream infection by data source.**

| **Data source** | **Outcome label** | **Central-line requirement** | **Blood-culture timing** | **Early infection handling** | **Event date** | **Secondary-source / contaminant handling** | **Interpretation** |
| --- | --- | --- | --- | --- | --- | --- | --- |
| MIMIC-IV | CLABSI-like bloodstream infection | Documented CVC exposure and ascertainable catheterisation time according to available database records | At least one positive blood culture obtained >48 h after catheter insertion during the post-catheterisation observation window | Blood-culture positivity within 48 h after catheter insertion was excluded | First qualifying positive blood culture | Formal secondary-source attribution and organism-specific contaminant adjudication were not uniformly available; this limitation was explicitly retained | Operational CLABSI-like BSI, not surveillance-confirmed CLABSI |
| eICU | CLABSI-like bloodstream infection | Documented CVC exposure and ascertainable catheterisation time according to available database records | At least one positive blood culture obtained >48 h after catheter insertion during the post-catheterisation observation window | Blood-culture positivity within 48 h after catheter insertion was excluded | First qualifying positive blood culture | Formal secondary-source attribution and organism-specific contaminant adjudication were not uniformly available; this limitation was explicitly retained | Operational CLABSI-like BSI, not surveillance-confirmed CLABSI |
| Chinese real-world cohort | Clinically adjudicated CLABSI | CVC exposure verified through hospital catheter insertion and removal records | Assessed according to routine CLABSI surveillance procedures and catheter-related timing rules | Early infections and pre-existing bloodstream infections were excluded during clinical adjudication | Adjudicated CLABSI event date recorded by the infection-control and clinical teams | Events were jointly reviewed by infectious disease/infection-control staff, the nursing department, the medical affairs department and treating clinicians; identifiable alternative bloodstream-infection sources, likely contaminants and non-CLABSI explanations were excluded | Surveillance-defined CLABSI adjudicated in routine hospital infection-control practice |

**Note. For the public databases, case ascertainment strictly followed variables available in MIMIC-IV and eICU and is therefore described as CLABSI-like bloodstream infection. The Chinese real-world cohort used clinically adjudicated CLABSI surveillance with multidisciplinary review.**

**Abbreviations: BSI, bloodstream infection; CLABSI, central line-associated bloodstream infection; CVC, central venous catheter; eICU, eICU Collaborative Research Database; MIMIC-IV, Medical Information Mart for Intensive Care IV.**

**Supplementary Table S2. Missingness of study variables across the final analytical cohorts.**

| **Variable** | **Variable type** | **MIMIC-IV (N = 10,868)** | **eICU-CRD (N = 1,489)** | **Chinese real-world cohort (N = 336)** |
| --- | --- | --- | --- | --- |
| ICU stay unique identifier | Identifier | 0 (0.00%) | — | — |
| Catheter type | Categorical | 0 (0.00%) | — | 0 (0.00%) |
| Catheter insertion site | Categorical | 0 (0.00%) | — | 0 (0.00%) |
| CLABSI outcome | Outcome | 0 (0.00%) | 0 (0.00%) | 0 (0.00%) |
| Sex | Categorical | 0 (0.00%) | 0 (0.00%) | 0 (0.00%) |
| Race | Categorical | 0 (0.00%) | — | — |
| Marital status | Categorical | 0 (0.00%) | — | — |
| Insurance type | Categorical | 0 (0.00%) | — | — |
| Mechanical ventilation | Categorical | 0 (0.00%) | 0 (0.00%) | 0 (0.00%) |
| Antibiotic exposure within 48 h before CVC insertion | Categorical | 0 (0.00%) | 0 (0.00%) | 0 (0.00%) |
| Vasopressor use | Categorical | 0 (0.00%) | 0 (0.00%) | 0 (0.00%) |
| Arterial catheter | Categorical | 0 (0.00%) | 0 (0.00%) | 0 (0.00%) |
| Dialysis | Categorical | 0 (0.00%) | 0 (0.00%) | 0 (0.00%) |
| Catheter duration, h | Continuous/count | 0 (0.00%) | 0 (0.00%) | 0 (0.00%) |
| Catheter duration, d | Continuous/count | 0 (0.00%) | 0 (0.00%) | 0 (0.00%) |
| Age | Continuous/count | 0 (0.00%) | 0 (0.00%) | 0 (0.00%) |
| Hospital length of stay, d | Continuous/count | 0 (0.00%) | 0 (0.00%) | 0 (0.00%) |
| ICU length of stay, d | Continuous/count | 4 (0.04%) | 0 (0.00%) | 0 (0.00%) |
| Admission weight | Continuous/count | 733 (6.74%) | 14 (0.94%) | 10 (2.98%) |
| Body mass index | Continuous/count | 2,879 (26.49%) | 653 (43.85%) | 160 (47.62%) |
| Ventilation duration before CVC insertion | Continuous/count | 0 (0.00%) | 0 (0.00%) | 72 (21.43%) |
| Mean temperature | Continuous/count | 809 (7.44%) | 206 (13.83%) | 46 (13.69%) |
| Maximum temperature | Continuous/count | 809 (7.44%) | 206 (13.83%) | 46 (13.69%) |
| Minimum temperature | Continuous/count | 809 (7.44%) | 206 (13.83%) | 46 (13.69%) |
| Mean heart rate | Continuous/count | 23 (0.21%) | 46 (3.09%) | 21 (6.25%) |
| Maximum heart rate | Continuous/count | 23 (0.21%) | 46 (3.09%) | 21 (6.25%) |
| Mean systolic blood pressure | Continuous/count | 48 (0.44%) | 53 (3.56%) | 27 (8.04%) |
| Mean diastolic blood pressure | Continuous/count | 839 (7.72%) | 53 (3.56%) | 27 (8.04%) |
| Mean arterial pressure | Continuous/count | 1,060 (9.75%) | 56 (3.76%) | 29 (8.63%) |
| Mean respiratory rate | Continuous/count | 24 (0.22%) | 68 (4.57%) | 25 (7.44%) |
| Mean oxygen saturation | Continuous/count | 23 (0.21%) | 77 (5.17%) | 28 (8.33%) |
| Minimum oxygen saturation | Continuous/count | 23 (0.21%) | 77 (5.17%) | 28 (8.33%) |
| Hematocrit | Continuous/count | 321 (2.95%) | 117 (7.86%) | 38 (11.31%) |
| Hemoglobin | Continuous/count | 355 (3.27%) | 139 (9.34%) | 39 (11.61%) |
| White blood cell count | Continuous/count | 356 (3.28%) | 139 (9.34%) | 39 (11.61%) |
| Platelet count | Continuous/count | 355 (3.27%) | 112 (7.52%) | 37 (11.01%) |
| Lactate | Continuous/count | 1,287 (11.84%) | 379 (25.45%) | 81 (24.11%) |
| Creatinine | Continuous/count | 279 (2.57%) | 165 (11.08%) | 44 (13.10%) |
| Blood urea nitrogen | Continuous/count | 283 (2.60%) | 168 (11.28%) | 44 (13.10%) |
| Sodium | Continuous/count | 888 (8.17%) | 351 (23.57%) | 58 (17.26%) |
| Potassium | Continuous/count | 640 (5.89%) | 387 (25.99%) | 62 (18.45%) |
| Calcium | Continuous/count | 2,211 (20.34%) | 837 (56.21%) | 128 (38.10%) |
| Prothrombin time | Continuous/count | 907 (8.35%) | 430 (28.88%) | 115 (34.23%) |
| Activated partial thromboplastin time | Continuous/count | 948 (8.72%) | 437 (29.35%) | 120 (35.71%) |
| International normalized ratio | Continuous/count | 906 (8.34%) | 431 (28.95%) | 115 (34.23%) |
| Glucose | Continuous/count | 1,663 (15.30%) | 509 (34.18%) | 73 (21.73%) |
| White blood cell count at 48 h after CVC insertion | Continuous/count | 50 (0.46%) | 38 (2.55%) | 11 (3.27%) |
| Baseline white blood cell count | Continuous/count | 1,466 (13.49%) | 46 (3.09%) | 11 (3.27%) |
| Vasopressor dose | Continuous/count | 0 (0.00%) | 0 (0.00%) | 0 (0.00%) |
| Daily EHR charting frequency | Continuous/count | 0 (0.00%) | 0 (0.00%) | 0 (0.00%) |
| Number of antibiotic agents | Continuous/count | 0 (0.00%) | 0 (0.00%) | 0 (0.00%) |
| Number of concurrent vascular lines | Continuous/count | 0 (0.00%) | 0 (0.00%) | 0 (0.00%) |
| Number of unique caregivers | Continuous/count | 0 (0.00%) | 0 (0.00%) | 0 (0.00%) |
| Fluid input within 24 h before CVC insertion | Continuous/count | 1,739 (16.00%) | 103 (6.92%) | 0 (0.00%) |
| Fluid input within 24 h after CVC insertion | Continuous/count | 1,739 (16.00%) | 103 (6.92%) | 0 (0.00%) |
| Fluid output within 24 h before CVC insertion | Continuous/count | 1,739 (16.00%) | 103 (6.92%) | 0 (0.00%) |
| Fluid output within 24 h after CVC insertion | Continuous/count | 1,739 (16.00%) | 103 (6.92%) | 0 (0.00%) |

**Note. Values are shown as n (%). Percentages were recalculated using the final analytical cohort denominators. Counts were retained as provided. An em dash indicates that the variable was not harmonised or not available in that data source. Missing score components were not imputed for score construction; the score was calculated as the mean of available robustly standardised indicators.**

**Abbreviations: CLABSI, central line-associated bloodstream infection; CVC, central venous catheter; eICU-CRD, eICU Collaborative Research Database; EHR, electronic health record; ICU, intensive care unit; MIMIC-IV, Medical Information Mart for Intensive Care IV; SpO2, peripheral oxygen saturation.**

**Supplementary Table S3. Availability of selected illness-severity, procedural and infection-prevention variables relevant to residual confounding.**

| **Variable or domain** | **MIMIC-IV** | **eICU** | **Chinese real-world cohort** |
| --- | --- | --- | --- |
| SOFA/APACHE/SAPS | Not harmonised in the current analysis file | Not harmonised in the current analysis file | Not harmonised in the current analysis file |
| Comorbidity burden | Not uniformly harmonised | Not uniformly harmonised | Not uniformly harmonised |
| Immunosuppression / malignancy | Not uniformly harmonised | Not uniformly harmonised | Not uniformly harmonised |
| Parenteral nutrition | Not uniformly harmonised | Not uniformly harmonised | Not uniformly harmonised |
| Catheter insertion site | Available | Not available | Available |
| Catheter type | Available | Not available | Available |
| Number of lumens | Not uniformly harmonised | Not uniformly harmonised | Not uniformly harmonised |
| Emergency versus elective insertion | Not uniformly harmonised | Not uniformly harmonised | Not uniformly harmonised |
| Unit-level workload / staffing | Not available at patient level | Not available at patient level | Not available at patient level |
| CLABSI bundle adherence | Not available | Not available | Not available |

**Note. Variables not uniformly available across all three data sources were not forced into the pooled primary models to avoid source-specific ascertainment bias.**

**Abbreviations: APACHE, Acute Physiology and Chronic Health Evaluation; CLABSI, central line-associated bloodstream infection; SAPS, Simplified Acute Physiology Score; SOFA, Sequential Organ Failure Assessment.**

**Supplementary Table S4. Distribution of care-exposure burden quartiles and CLABSI or CLABSI-like events by data source.**

| **Database** | **Quartile** | **Score range** | **Median (IQR)** | **n** | **Events** | **Event rate (%)** |
| --- | --- | --- | --- | --- | --- | --- |
| MIMIC-IV | Q1 lowest | -2.962 to -0.216 | -0.358 (-0.499, -0.281) | 2,717 | 28 | 1.0 |
| MIMIC-IV | Q2 | -0.216 to -0.028 | -0.116 (-0.165, -0.072) | 2,717 | 37 | 1.4 |
| MIMIC-IV | Q3 | -0.027 to 0.178 | 0.070 (0.019, 0.120) | 2,717 | 97 | 3.6 |
| MIMIC-IV | Q4 highest | 0.178 to 1.242 | 0.319 (0.242, 0.433) | 2,717 | 350 | 12.9 |
| eICU | Q1 lowest | -2.362 to -0.269 | -0.474 (-0.662, -0.347) | 373 | 54 | 14.5 |
| eICU | Q2 | -0.269 to -0.062 | -0.165 (-0.218, -0.116) | 372 | 51 | 13.7 |
| eICU | Q3 | -0.061 to 0.162 | 0.043 (-0.002, 0.107) | 372 | 78 | 21.0 |
| eICU | Q4 highest | 0.164 to 0.805 | 0.307 (0.233, 0.409) | 372 | 102 | 27.4 |
| Chinese real-world cohort | Q1 lowest | -1.732 to -0.229 | -0.433 (-0.644, -0.304) | 84 | 22 | 26.2 |
| Chinese real-world cohort | Q2 | -0.227 to -0.014 | -0.120 (-0.181, -0.070) | 84 | 20 | 23.8 |
| Chinese real-world cohort | Q3 | -0.010 to 0.206 | 0.084 (0.040, 0.128) | 84 | 31 | 36.9 |
| Chinese real-world cohort | Q4 highest | 0.208 to 1.248 | 0.425 (0.326, 0.513) | 84 | 40 | 47.6 |

**Note. Quartiles were defined according to the care-exposure burden distribution within each data source. Event rates are shown within each quartile.**

**Abbreviations: CLABSI, central line-associated bloodstream infection; IQR, interquartile range.**

**Supplementary Table S5. Source-specific associations between care-exposure burden quartiles and CLABSI or CLABSI-like bloodstream infection.**

| **Data source** | **Exposure contrast** | **Adjusted OR (95% CI)** | **P value** |
| --- | --- | --- | --- |
| MIMIC-IV | Care-exposure burden: Q2 vs Q1 | 1.53 (0.83–2.90) | 0.175 |
| MIMIC-IV | Care-exposure burden: Q3 vs Q1 | 3.23 (1.93–5.72) | <0.001 |
| MIMIC-IV | Care-exposure burden: Q4 vs Q1 | 7.68 (4.77–13.22) | <0.001 |
| eICU | Care-exposure burden: Q2 vs Q1 | 1.20 (0.72–2.00) | 0.488 |
| eICU | Care-exposure burden: Q3 vs Q1 | 1.40 (0.88–2.24) | 0.157 |
| eICU | Care-exposure burden: Q4 vs Q1 | 1.54 (0.98–2.47) | 0.065 |
| Chinese real-world cohort | Care-exposure burden: Q2 vs Q1 | 0.95 (0.45–2.00) | 0.893 |
| Chinese real-world cohort | Care-exposure burden: Q3 vs Q1 | 1.78 (0.89–3.62) | 0.104 |
| Chinese real-world cohort | Care-exposure burden: Q4 vs Q1 | 2.49 (1.25–5.07) | 0.010 |

**Note. Separate regression models were fitted within each data source. Q1 was used as the reference category.**

**Abbreviations: CI, confidence interval; OR, odds ratio.**

**Supplementary Table S6. Distribution of catheter type and insertion site by CLABSI status in data sources with available catheter-characteristic information.**

| **Characteristic** | **No CLABSI** | **CLABSI** |
| --- | --- | --- |
| Data source |  |  |
| MIMIC-IV | 10,356 (97.9%) | 512 (81.9%) |
| Chinese real-world cohort | 223 (2.1%) | 113 (18.1%) |
| Catheter type |  |  |
| Type 1 | 2 (0.0%) | 0 (0.0%) |
| Type 2 | 106 (1.0%) | 33 (5.3%) |
| Type 3 | 26 (0.2%) | 4 (0.6%) |
| Type 4 | 70 (0.7%) | 2 (0.3%) |
| Type 5 | 3,134 (29.6%) | 37 (5.9%) |
| Type 6 | 7,241 (68.4%) | 549 (87.8%) |
| Insertion site |  |  |
| Site 1 | 6,753 (63.8%) | 270 (43.2%) |
| Site 2 | 1,112 (10.5%) | 56 (9.0%) |
| Site 3 | 968 (9.2%) | 130 (20.8%) |
| Site 4 | 689 (6.5%) | 75 (12.0%) |
| Site 5 | 544 (5.1%) | 72 (11.5%) |
| Site 6 | 503 (4.8%) | 22 (3.5%) |
| Site 7 | 8 (0.1%) | 0 (0.0%) |
| Site 8 | 1 (0.0%) | 0 (0.0%) |
| Site 9 | 1 (0.0%) | 0 (0.0%) |

**Note. Values are n (%). eICU was not included because catheter type and insertion site were not available in harmonised form. Category labels reflect de-identified source categories.**

**Abbreviations: CLABSI, central line-associated bloodstream infection.**

**Supplementary Table S7. Linear trend in CLABSI or CLABSI-like bloodstream infection across care-exposure burden quartiles.**

| **Model specification** | **Exposure** | **OR per quartile increase (95% CI)** | **P for trend** |
| --- | --- | --- | --- |
| Burden quartile modelled as an ordinal variable | Care-exposure burden quartile (Q1–Q4) | 1.78 (1.62–1.96) | <0.001 |

**Note. Care-exposure burden quartile was modelled as an ordinal variable from Q1 to Q4.**

**Abbreviations: CI, confidence interval; OR, odds ratio.**

**Supplementary Table S8. Discrimination and calibration diagnostics of multivariable regression models.**

| **Model** | **AUC** | **Brier score** |
| --- | --- | --- |
| Model 1: data source + age/sex | 0.808 | 0.06 |
| Model 2: additionally adjusted for clinical status | 0.813 | 0.072 |
| Model 3: domain scores | 0.843 | 0.07 |

**Note. Diagnostics are reported only to describe model behaviour in this retrospective association study and should not be interpreted as development or validation of a clinical prediction model.**

**Abbreviations: AUC, area under the receiver operating characteristic curve.**

**Supplementary Table S9. Firth penalised logistic regression sensitivity analysis using Model 2 covariates.**

| **Group** | **Variable** | **Adjusted OR (95% CI)** | **P value** |
| --- | --- | --- | --- |
| Primary exposure | Care-exposure burden: Q2 vs Q1 | 1.34 (0.91–1.96) | 0.135 |
| Primary exposure | Care-exposure burden: Q3 vs Q1 | 2.42 (1.73–3.42) | <0.001 |
| Primary exposure | Care-exposure burden: Q4 vs Q1 | 4.79 (3.51–6.65) | <0.001 |
| Database indicators | Database: eICU vs MIMIC-IV | 9.67 (1.06–90.55) | 0.043 |
| Database indicators | Database: Chinese real-world cohort vs MIMIC-IV | 24.56 (2.67–231.49) | 0.002 |
| Covariates | Age | 0.98 (0.98–0.99) | <0.001 |
| Covariates | Male sex | 1.27 (1.07–1.52) | 0.008 |
| Covariates | Mean temperature, °C | 1.01 (0.98–1.05) | 0.601 |
| Covariates | Mean heart rate | 1.00 (1.00–1.01) | 0.268 |
| Covariates | Mean arterial pressure | 1.00 (0.99–1.01) | 0.645 |
| Covariates | Mean respiratory rate | 1.04 (1.01–1.06) | 0.001 |
| Covariates | Minimum SpO2 | 1.00 (0.99–1.00) | 0.203 |
| Covariates | Baseline WBC | 1.01 (1.00–1.01) | 0.131 |
| Covariates | Lactate | 1.02 (0.99–1.05) | 0.208 |
| Covariates | Creatinine | 1.01 (0.95–1.07) | 0.823 |
| Covariates | Blood urea nitrogen | 1.01 (1.01–1.02) | <0.001 |
| Covariates | Platelet count | 1.00 (1.00–1.00) | <0.001 |
| Covariates | Hemoglobin | 1.01 (0.96–1.05) | 0.769 |
| Covariates | Glucose | 1.00 (1.00–1.00) | 0.869 |

**Note. Firth penalised logistic regression was used as a small-sample and separation-robust sensitivity analysis. The intercept was omitted.**

**Abbreviations: CI, confidence interval; eICU, eICU Collaborative Research Database; MIMIC-IV, Medical Information Mart for Intensive Care IV; OR, odds ratio; SpO2, peripheral oxygen saturation; WBC, white blood cell count.**

**Supplementary Table S10. Sensitivity analysis additionally adjusted for ICU and hospital length of stay.**

| **Variable** | **Adjusted OR (95% CI)** | **P value** |
| --- | --- | --- |
| Care-exposure burden: Q2 vs Q1 | 1.30 (0.88–1.92) | 0.194 |
| Care-exposure burden: Q3 vs Q1 | 1.95 (1.38–2.79) | <0.001 |
| Care-exposure burden: Q4 vs Q1 | 2.40 (1.72–3.40) | <0.001 |
| Database: eICU vs MIMIC-IV | 5.77 (0.76–53.02) | 0.128 |
| Database: Chinese real-world cohort vs MIMIC-IV | 13.07 (1.70–120.86) | 0.026 |
| Mean temperature, °C | 0.98 (0.98–0.99) | <0.001 |
| Mean heart rate | 1.30 (1.08–1.57) | 0.005 |
| Mean arterial pressure | 1.00 (0.97–1.04) | 0.956 |
| Mean respiratory rate | 1.01 (1.00–1.01) | 0.115 |
| Minimum SpO2 | 1.00 (0.99–1.00) | 0.219 |
| Baseline WBC | 1.02 (1.00–1.04) | 0.126 |
| Lactate | 1.00 (1.00–1.01) | 0.919 |
| Creatinine | 1.00 (0.99–1.01) | 0.515 |
| Blood urea nitrogen | 1.02 (0.99–1.05) | 0.204 |
| Platelet count | 1.00 (0.94–1.06) | 0.970 |
| Hemoglobin | 1.01 (1.01–1.01) | <0.001 |
| Glucose | 1.00 (1.00–1.00) | 0.019 |
| Mean temperature, °C | 1.00 (0.96–1.05) | 0.965 |
| Mean heart rate | 1.00 (1.00–1.00) | 0.860 |
| Hospital length of stay, days | 1.00 (0.99–1.00) | 0.810 |
| ICU length of stay, days | 1.07 (1.06–1.08) | <0.001 |

**Note. Length-of-stay variables were treated as sensitivity covariates rather than primary covariates because they may lie on the causal or post-outcome pathway.**

**Abbreviations: CI, confidence interval; ICU, intensive care unit; OR, odds ratio.**

**Supplementary Table S11. E-value analysis for potential unmeasured confounding.**

| **Model** | **Contrast** | **Adjusted OR** | **95% CI limit closest to the null** | **E-value for point estimate** | **E-value for CI limit** |
| --- | --- | --- | --- | --- | --- |
| Model 2 | Care-exposure burden Q4 highest vs Q1 lowest | 4.87 | 3.56 | 9.2 | 6.58 |

**Note. The E-value estimates the minimum strength of association that an unmeasured confounder would need to have with both the exposure and outcome, conditional on measured covariates, to explain away the observed association.**

**Abbreviations: CI, confidence interval; OR, odds ratio.**

**Supplementary Table S12. Components of the 48-hour landmark-compatible care-exposure burden score excluding total catheter duration.**

| **Score component** | **Included in no-duration score** | **Timing interpretation** | **Reason for inclusion/exclusion** |
| --- | --- | --- | --- |
| Daily EHR charting frequency | Yes | Fixed early/landmark-compatible EHR exposure indicator | Documentation/contact intensity |
| Number of unique caregivers | Yes | Fixed early/landmark-compatible EHR exposure indicator | Care-team complexity |
| Number of concurrent vascular lines | Yes | Fixed early/landmark-compatible EHR exposure indicator | Concurrent device burden |
| Ventilation duration before CVC insertion | Yes | Pre-insertion variable | Treatment complexity before line placement |
| Antibiotic-type count | Yes | Pre-specified antibiotic exposure indicator | Treatment complexity / antimicrobial exposure |
| Fluid input within 24 h after CVC insertion | Yes | Available before 48-h landmark | Early fluid-management complexity |
| Fluid output within 24 h after CVC insertion | Yes | Available before 48-h landmark | Early fluid-management complexity |
| Total catheter duration | No | Post-landmark/cumulative follow-up variable | Excluded because duration up to 48 h is fixed by design among landmark-eligible patients, whereas total duration contains post-landmark information |

**Note. This sensitivity score used components available within the fixed early post-catheterisation window or before catheter insertion. Total catheter duration was excluded because catheter duration up to 48 hours was fixed by design among landmark-eligible patients, whereas total duration contained post-landmark information.**

**Abbreviations: CVC, central venous catheter; EHR, electronic health record.**

**Supplementary Table S13. Distribution of the landmark-compatible score excluding catheter duration and event rates by data source.**

| **Data source** | **Quartile** | **n** | **CLABSI/CLABSI-like events** | **Event rate (%)** | **Score range** | **Median (IQR)** |
| --- | --- | --- | --- | --- | --- | --- |
| MIMIC-IV | Q1 lowest | 2,717 | 46 | 1.7 | -3.651 to -0.222 | -0.370 (-0.526, -0.285) |
| MIMIC-IV | Q2 | 2,717 | 53 | 2.0 | -0.222 to -0.024 | -0.116 (-0.164, -0.069) |
| MIMIC-IV | Q3 | 2,717 | 105 | 3.9 | -0.024 to 0.165 | 0.066 (0.017, 0.112) |
| MIMIC-IV | Q4 highest | 2,717 | 308 | 11.3 | 0.165 to 1.403 | 0.290 (0.223, 0.397) |
| eICU | Q1 lowest | 373 | 66 | 17.7 | -2.502 to -0.282 | -0.512 (-0.724, -0.384) |
| eICU | Q2 | 372 | 60 | 16.1 | -0.282 to -0.071 | -0.165 (-0.221, -0.116) |
| eICU | Q3 | 372 | 70 | 18.8 | -0.070 to 0.139 | 0.034 (-0.015, 0.087) |
| eICU | Q4 highest | 372 | 89 | 23.9 | 0.140 to 0.918 | 0.303 (0.222, 0.394) |
| Chinese real-world cohort | Q1 lowest | 84 | 26 | 31.0 | -1.737 to -0.257 | -0.413 (-0.715, -0.327) |
| Chinese real-world cohort | Q2 | 84 | 24 | 28.6 | -0.257 to -0.001 | -0.100 (-0.167, -0.043) |
| Chinese real-world cohort | Q3 | 84 | 26 | 31.0 | 0.001 to 0.209 | 0.099 (0.047, 0.149) |
| Chinese real-world cohort | Q4 highest | 84 | 37 | 44.0 | 0.217 to 1.210 | 0.408 (0.315, 0.538) |

**Note. Quartiles were defined within each data source after excluding total catheter duration from the composite score.**

**Abbreviations: CLABSI, central line-associated bloodstream infection; IQR, interquartile range.**

**Supplementary Table S14. Pooled adjusted models using the landmark-compatible score excluding total catheter duration.**

| **Model** | **Label** | **Adjusted OR (95% CI)** | **P value** |
| --- | --- | --- | --- |
| Model 1 Database + age/sex | Q2 vs Q1 | 0.98 (0.77–1.26) | 0.901 |
| Model 1 Database + age/sex | Q3 vs Q1 | 1.52 (1.21–1.92) | <0.001 |
| Model 1 Database + age/sex | Q4 vs Q1 | 3.68 (2.99–4.52) | <0.001 |
| Model 2 Clinical adjustment | Q2 vs Q1 | 1.46 (1.05–2.04) | 0.024 |
| Model 2 Clinical adjustment | Q3 vs Q1 | 1.77 (1.30–2.41) | <0.001 |
| Model 2 Clinical adjustment | Q4 vs Q1 | 3.20 (2.40–4.25) | <0.001 |
| Model 3 Domain-score model | Documentation/contact score | 2.19 (1.79–2.68) | <0.001 |
| Model 3 Domain-score model | Catheter/device score (no duration) | 0.68 (0.49–0.94) | 0.019 |
| Model 3 Domain-score model | Treatment-complexity score | 1.92 (1.58–2.34) | <0.001 |

**Note. Model 1 adjusted for data source, age and sex. Model 2 additionally adjusted for available clinical status variables. Model 3 used domain scores recalculated without total catheter duration.**

**Abbreviations: CI, confidence interval; OR, odds ratio.**

**Supplementary Table S15. Source-specific associations using the landmark-compatible score excluding total catheter duration.**

| **Data source** | **Contrast** | **Adjusted OR (95% CI)** | **P value** |
| --- | --- | --- | --- |
| MIMIC-IV | Q2 vs Q1 | 1.70 (1.02–2.81) | 0.041 |
| MIMIC-IV | Q3 vs Q1 | 2.49 (1.57–3.96) | <0.001 |
| MIMIC-IV | Q4 vs Q1 | 5.07 (3.30–7.78) | <0.001 |
| eICU | Q2 vs Q1 | 1.13 (0.70–1.81) | 0.622 |
| eICU | Q3 vs Q1 | 1.06 (0.68–1.65) | 0.796 |
| eICU | Q4 vs Q1 | 1.02 (0.66–1.59) | 0.919 |
| Chinese real-world cohort | Q2 vs Q1 | 0.99 (0.49–2.00) | 0.971 |
| Chinese real-world cohort | Q3 vs Q1 | 1.08 (0.54–2.17) | 0.829 |
| Chinese real-world cohort | Q4 vs Q1 | 1.63 (0.83–3.21) | 0.159 |

**Note. Separate models were fitted within each data source using the modified score excluding total catheter duration. Q1 was used as the reference category.**

**Abbreviations: CI, confidence interval; OR, odds ratio.**

**Supplementary Figures**

**Supplementary Figure S1. Heatmap of standardised care-exposure components by CLABSI status and data source.**


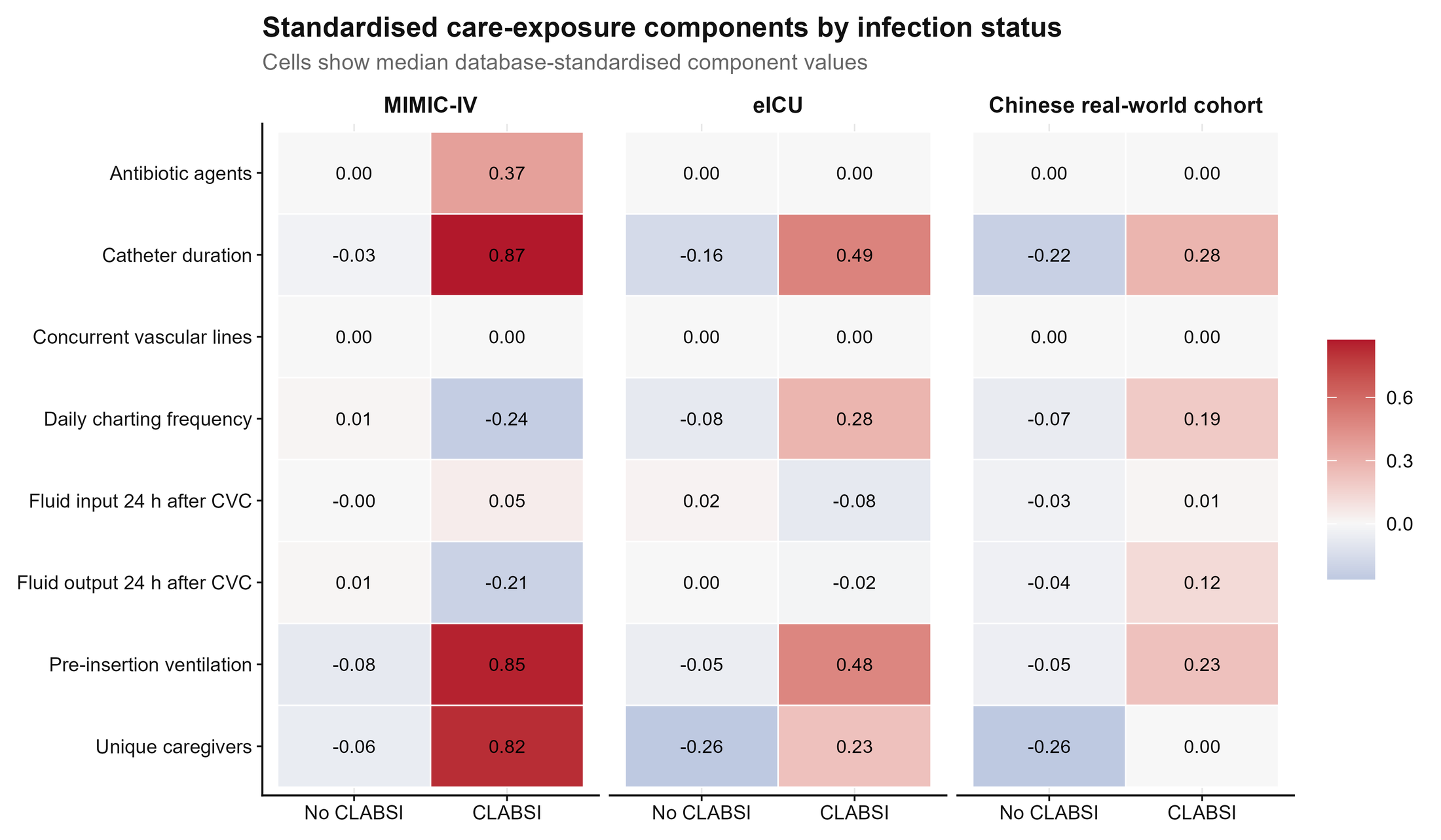


**Legend. Each cell shows the median standardised value within the corresponding subgroup. Warmer colours indicate higher median values and cooler colours indicate lower median values.**

**Supplementary Figure S2. Observed CLABSI or CLABSI-like event rates across deciles of care-exposure burden by data source.**


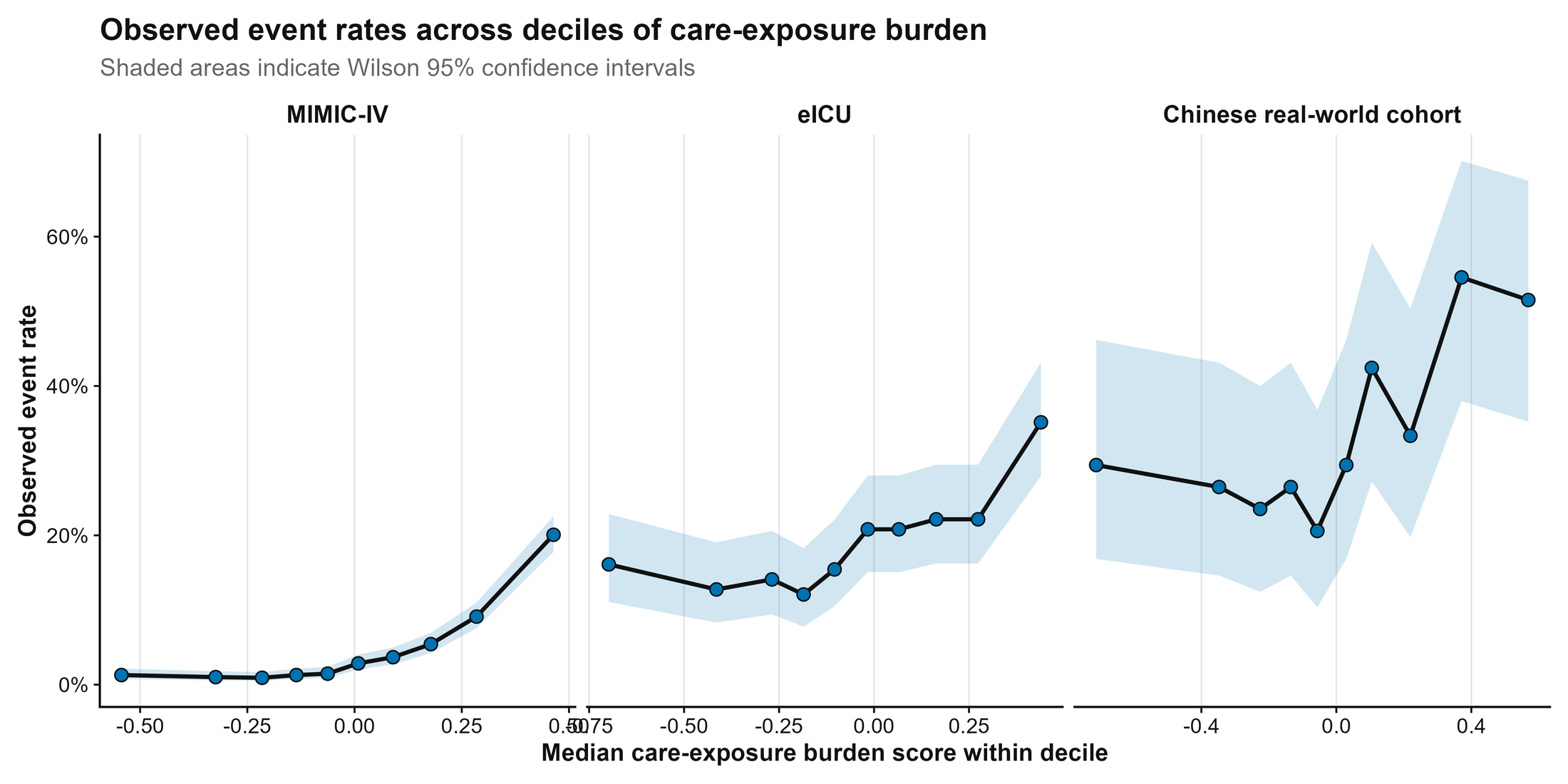


**Legend. Points represent observed event rates within deciles of the primary care-exposure burden score. Shaded areas represent Wilson 95% confidence intervals.**

**Supplementary Figure S3. Adjusted associations using the landmark-compatible score excluding total catheter duration.**


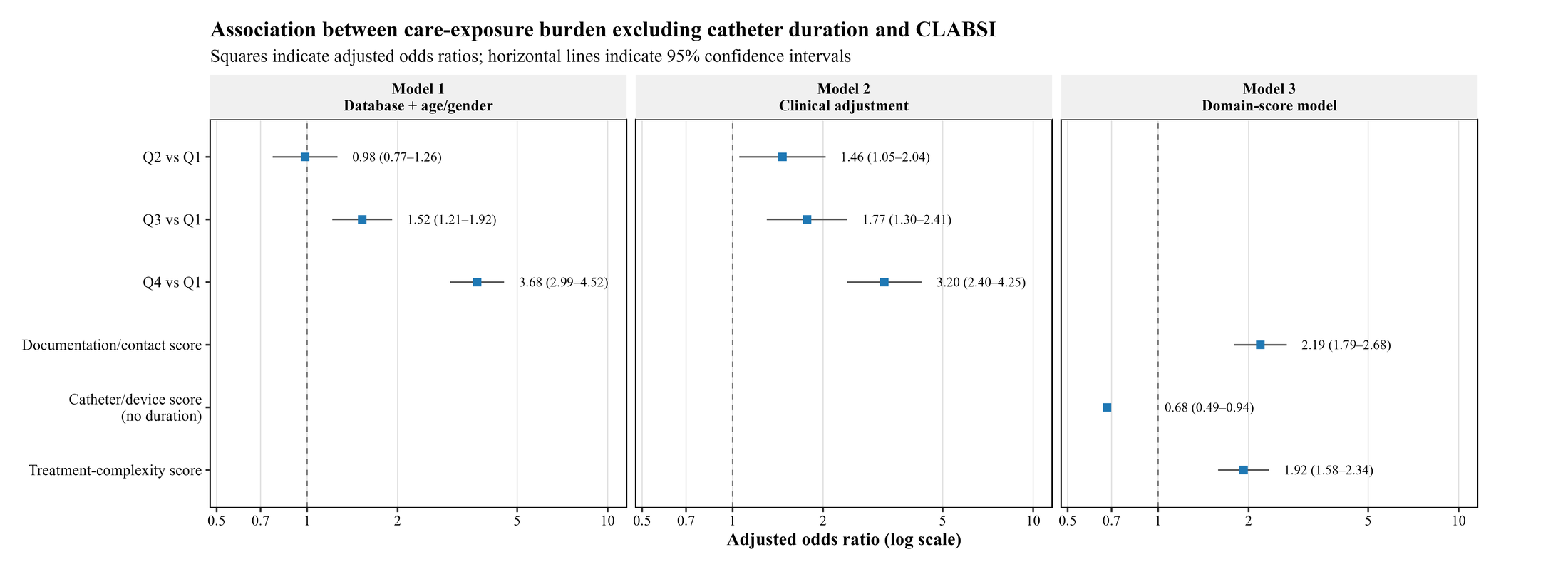


**Legend. The forest plot follows the main Figure 4 layout. Squares indicate adjusted odds ratios and horizontal lines indicate 95% confidence intervals. Model 1 adjusted for data source, age and sex; Model 2 additionally adjusted for available clinical status variables; Model 3 used domain scores recalculated without total catheter duration.**

**Supplementary Figure S4. Source-specific associations using the landmark-compatible score excluding total catheter duration.**


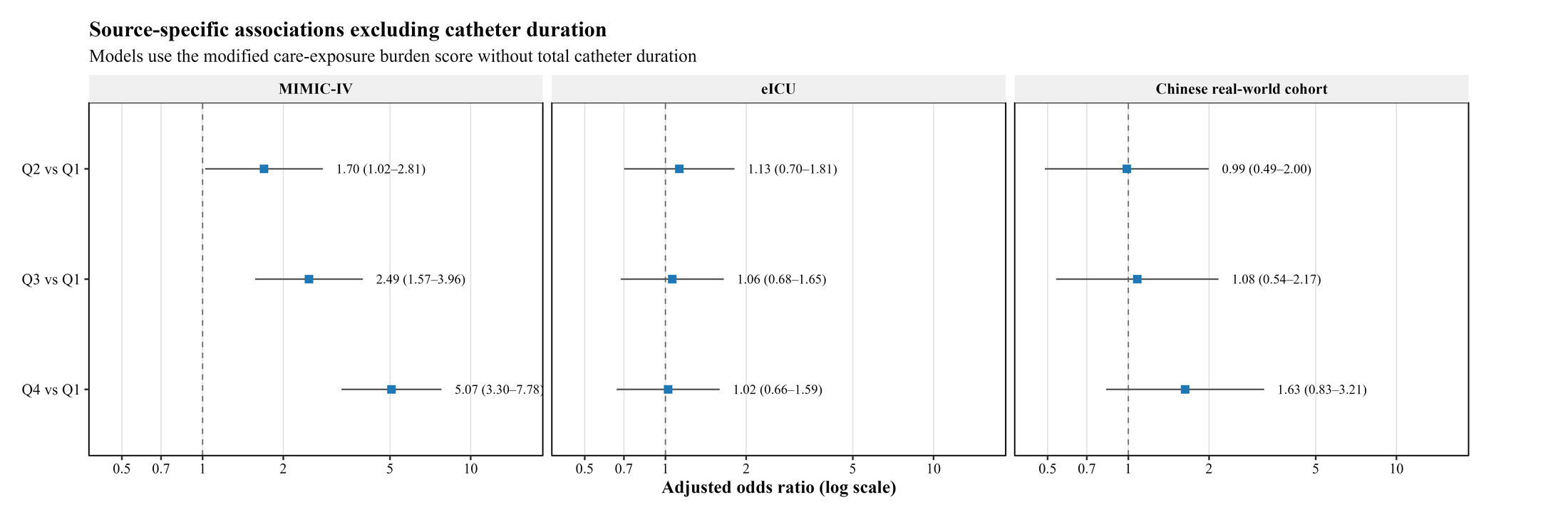


**Legend. Separate models were fitted within each data source. Squares indicate adjusted odds ratios and horizontal lines indicate 95% confidence intervals. Q1 was the reference category.**

**Supplementary Figure S5. Sensitivity analysis additionally adjusted for ICU and hospital length of stay.**


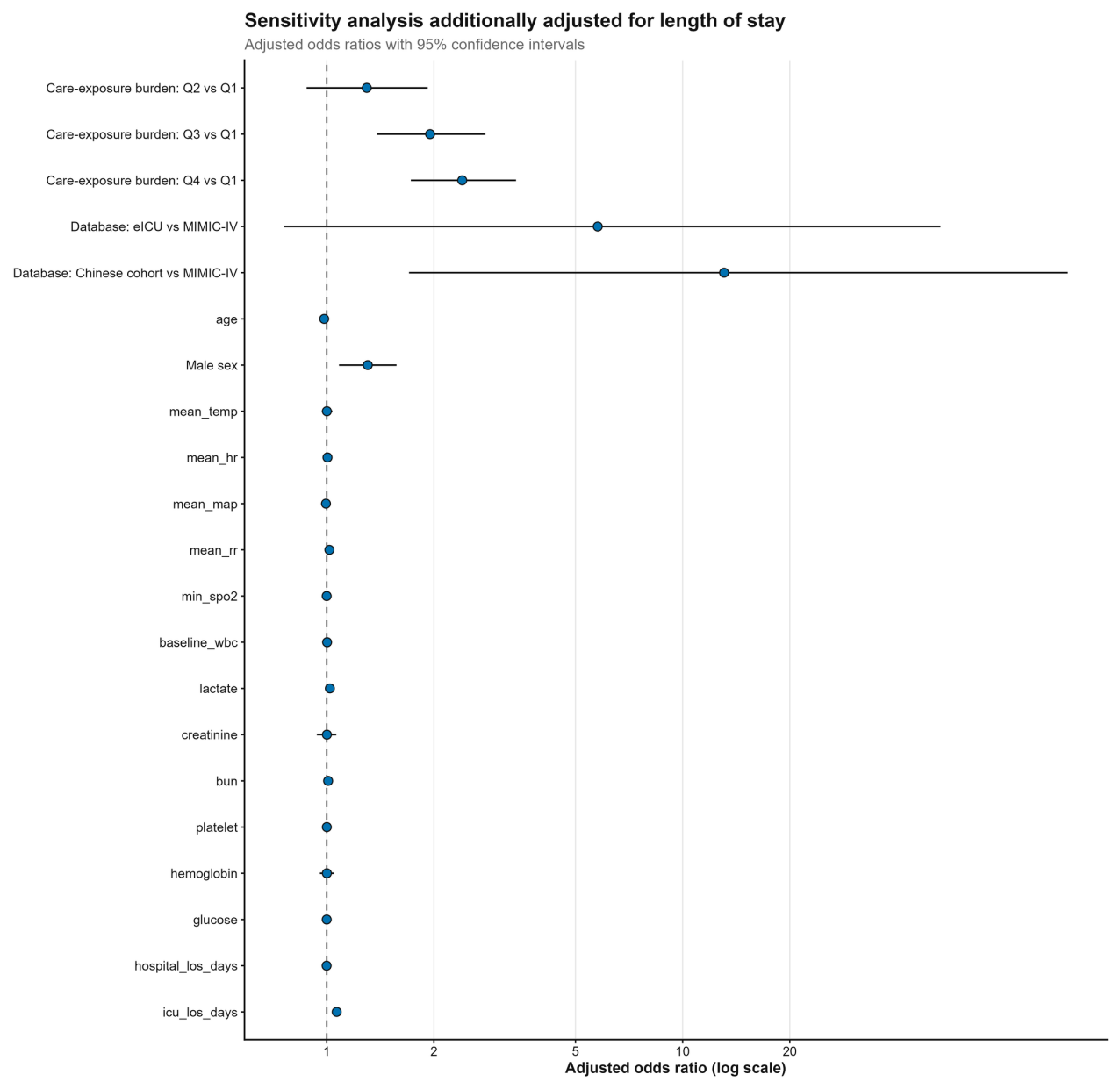


**Legend. The forest plot shows adjusted odds ratios with 95% confidence intervals. Length-of-stay variables were treated as sensitivity covariates rather than primary covariates.**
